# Beta diversity determinants in Badagongshan, a subtropical forest in central China

**DOI:** 10.1038/srep17043

**Published:** 2015-11-23

**Authors:** Xiujuan Qiao, Qianxi Li, Qinghu Jiang, Junmeng Lu, Scott Franklin, Zhiyao Tang, Qinggang Wang, Jiaxin Zhang, Zhijun Lu, Dachuan Bao, Yili Guo, Haibo Liu, Yaozhan Xu, Mingxi Jiang

**Affiliations:** 1 Key Laboratory of Aquatic Botany and Watershed Ecology, Wuhan Botanical Garden, Chinese Academy of Sciences, Wuhan 430074, P.R. China; 2 University of Chinese Academy of Sciences, Beijing 100049, P.R. China; 3 Department of Ecology, College of Urban and Environmental Sciences, Center for Ecological Research and Education, and Key Laboratory for Earth Surface Processes of the Ministry of Education, Peking University, Beijing 100871, P.R. China; 4 Department of Biological Sciences, University of Northern Colorado, Greeley, CO 80639, USA

## Abstract

Niche and neutral theories emphasize different processes contributing to the maintenance of species diversity. In this study, we calculated the local contribution to beta diversity (LCBD) of every cell, using variation partitioning in combination with spatial distance and environmental variables of the 25-ha Badagongshan plot (BDGS), to determine the contribution of environmentally-related variation versus pure spatial variation. We used topography and soil characteristics as environmental variables, distance-based Moran’s eigenvectors maps (dbMEM) to describe spatial relationships among cells and redundancy analysis (RDA) to apportion the variation in beta diversity into three components: pure environmental, spatially-structured environmental, and pure spatial. Results showed LCBD values were negatively related to number of common species and positively related to number of rare species. Environment and space jointly explained ~60% of the variation in species composition; soil variables alone explained 21.6%, slightly more than the topographic variables that explained 15.7%; topography and soil together explained 27%, slightly inferior to spatial variables that explained 34%. The BDGS forest was controlled both by the spatial and environmental variables, and the results were consistent across different life forms and life stages.

With the rapid loss of species during the sixth mass extinction, conserving biodiversity is extremely important^1^,^2^, and that requires an understanding of the mechanisms through which species are able to coexist. Niche and neutral theories emphasize different processes contributing to the maintenance of species diversity^3^,^4^,^5^. Niche theory proposes that, in a community at equilibrium, each species must occupy a different niche, emphasizing the importance of environmental filtering or species interactions in determining species composition^6^,^7^; neutral theory contrarily, assumes spatial processes alone determine species composition^8^,^9^,^10^. Beta diversity denotes the variation in species composition among sites in the geographic area of interest^11^ and is a key concept for understanding the processes that create and maintain biodiversity^7^,^12^,^13^. According to niche and neutral theories, processes generating beta diversity can be roughly grouped into two types^13^, environmental filtering^14^,^15^,^16^ and dispersal limitation^17^,^18^. Under environmental filtering, species distributions are controlled by the match of a species niche and environmental conditions^19^,^20^, so sites with similar conditions should harbour similar species^21^. Under dispersal limitation, geographic distance governs whether a species is present or absent from a site, and thus sites that are closer together should harbour more similar species composition^10^,^17^. Many empirical studies have shown the independent effects of environmental filtering and dispersal limitation on community assembly^22^,^23^,^24^,^25^, and generally, environmental filtering is the main driver of species aggregations in temperate forest, whereas dispersal limitation has a higher explanation power than environmental variables in the tropics^26^. However, most of these studies focused on the species turnover in a large region (for example, all of North American or China) with only coarse climatic data as environmental variables, which may not reflect the controlling factors of beta diversity in a local community.

In recent years, with the establishment of large permanent forest plots that include precise stem maps, detailed topography and detailed soil characteristics, it is possible to study local factors influencing beta diversity^27^,^28^,^29^,^30^. In a subtropical broadleaf forest in China, beta diversity was equally governed by environmental and neutral (i.e., dispersal) variables^30^, although only topography was included in this study as an environmental variable. A similar study in Lienhuachih (Taiwan) showed that dispersal limitation prevailed except when soil variables were included and then niche processes were more important^27^. So the quality of environmental data strongly affects the results^31^. Further, the relative effects of spatial and environmental factors on tree diversity patterns may also depend on the life stage of the trees considered^29^. For example, seedlings are generally more aggregated than adults because of the seedlings’ lack of relationship with environmental gradients^32^. In addition, life form of the focal species may affect the relative importance of different factors^33^. Many studies have suggested that understory species distributions are more dependent on the environment than canopy species distributions^34^.

Partitioning the variation in community structure among sampling units between environmental and spatial components provides a useful method to detect mechanisms underlying community assembly^7^,^13^,^35^. If beta diversity is strongly correlated with environmental variables, processes associated with environmental filtering are likely operating; otherwise, spatial processes (e.g., dispersal limitation) are likely playing a stronger role. The unexplained proportion by environment and space may be due to local stochasticity^30^, unmeasured environmental and spatial variables^36^ or sampling error. Our study was based on the detailed information of species abundance and distribution, topography and soil characteristics in the 25-ha forest plot located in the Badagongshan National Nature Reserve in Hunan Province, China. We partitioned the variations of species composition respectively explained by topography, soil and spatial variables to determine:

1. The beta diversity pattern across the plot;
2. The relationship of environmental variables versus pure spatial variables to tree beta diversity at the scale of the forest plot; and
3. Differences in these relationships among life stages and tree layers.

## Results

### Variation partitioning of community composition

**Figure 1:**
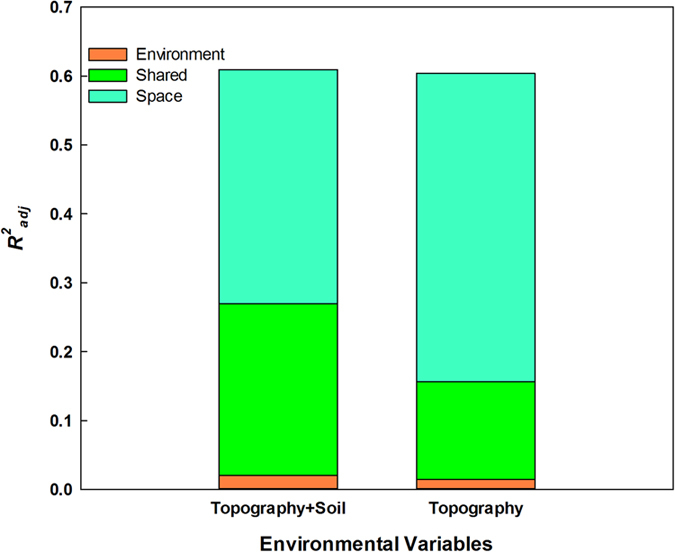
Variation partitioning of community composition into fractions explained by environmental and spatial variables reported as 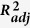.

For all trees, environmental (including topographic and soil) and spatial variables together explained 60.7% of the variation in community composition in the BDGS plot (Fig. 1). Topographic and soil variables together explained 27.0% and the spatial variables explained 34.0% of the variation in community composition, respectively. However, environmental variables alone explained little of the variation in community composition (i.e., most was spatially structured; Fig. 1).

**Figure 2:**
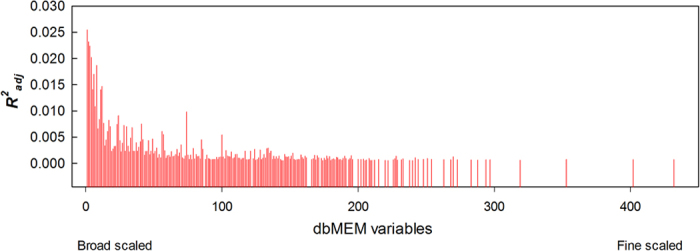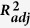 values of the 226 eigenfunctions selected by forward selection among the 440 variables generated by dbMEM. The dbMEM variables were sorted from broad-scaled to fine-scaled along the X-axis (from left to right), and only dbMEM variables with positive 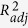 values are presented.

Forward selection retained 226 of the 440 dbMEM eigenfunctions modelling community composition, and the model R^2^_a_ was 58.9%, nearly equivalent to the value without selection, R^2^_a_ = 64.6%. Most of the variation was explained with the first 250 of the 440 dbMEM eigenfunctions (Fig. 2), which represented broad to middle-scale variation.

In order to compare with former studies, we reran the analysis separating topography from other environmental variables. The unexplained variation was unaffected by selection of environmental variables. Topographical and spatial variables together explained 60.4% of the variation in community composition in the BDGS plot, nearly equal to the full model (Fig. 1). However, the composition of the explained variations was different. Only 15.7% of community composition was explained by topographic variables, and the variation explained by the pure spatial component increased from 34.0% to 44.7%. From all of the above, we can deduce that soil variables increase the explanatory power of environmental variables to some extent. Thus, the following results of variation partitioning for different types of trees include both topography and soil as environmental variables.

### Variation partitioning of different life forms

**Figure 3:**
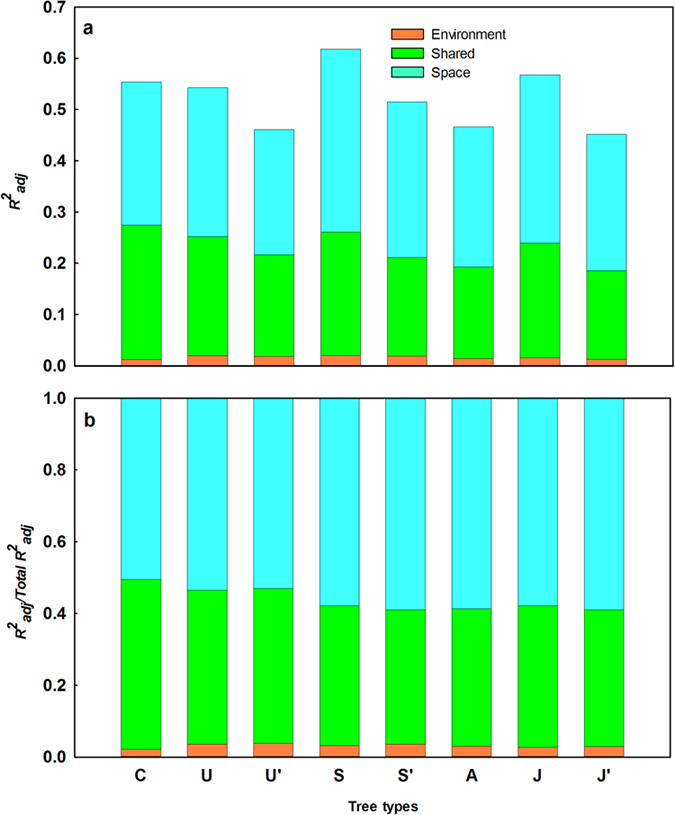
Variation partitioning of community composition into fractions explained by environment and space for different life stages and tree layers. (**a**) the absolute fractions of variation explained; (**b**) the relative fractions of variation explained in relation to the total variation explained. Each bar represents a group: C = canopy trees; U = Understory trees; U’ = Understory trees after removing sampling effects; S = shrubs; S’ = shrubs after removing sampling effects; A = adults; J = juveniles; J’ = juveniles after removing sampling effects. The reported fractions are 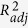.

Spatial and environmental variables together explained more variation in shrubs (61.8%) than in canopy (55.4%) or understory trees (54.3%). However, after removing sampling effects, i.e., after accounting for the different number of individuals sampled in the three categories, the explanatory strength of the models decreased to 51.5% for shrubs and 46.1% for understory trees (Fig. 3a). Canopy trees had the highest total variation explained and were more related to environmental variables (27.4% vs 21.6% for understory trees and 21.1% for shrubs after removing sampling effects).

Among different tree layers, pure spatial effects explained more variation than environment for canopy (28.0% vs. 27.4%), understory (29.0% vs. 25.2%) and shrub (35.8% vs. 26.1%) individuals. For further comparison, we calculated the relative percentage of total explained variation of environment and space to different life forms. The relative fraction of variation explained by the environment was highest for canopy trees (49.5%), followed by understory, for which it accounted for 46.5% of explained variation (46.9% after removing sampling effects), and shrubs, for which it accounted for 42.1% of explained variation (41.0% after removing sampling effects) (Fig. 3b).

### Variation partitioning of different life stages

The total variation explained was higher for juvenile (56.8%) than for adult trees (46.6%). However, after controlling for sampling effects by only keeping as many individuals as the adults in the analysis, the explained variation for juvenile trees was reduced to 45.2%, equivalent to that of adult trees. Consistent with the results for all trees and different life forms, pure spatial effects explained more variation than environment for both adult (27.3% vs. 19.3%) and juvenile trees (32.8% vs. 24.0%) (Fig. 3a). The relative contribution to the total variation explained by the environment and space were respectively 41.3% and 58.7% for the adults and 42.2% and 57.8% for the juveniles (41.1% and 58.9% after removing sampling effects) (Fig. 3b).

### Local contributions to beta diversity

The local contributions to beta diversity ranged from 0.0021 to 0.0037, and beta diversity was significantly related to all topographic variables, indicating topography significantly affected species turnover in this plot (Table 1). Only three out of nine soil parameters of the upper soil layer were significantly related to beta diversity, i.e., δ^13^C, N/P ratio and pH value. Four of the five indices of the lower soil were significantly related to beta diversity as well, including δ^13^C, N content, P contents and N/P ratio. pH values had the strongest relationship with LCBD with an R_a_^2^ of 0.159.

**Figure 4:**
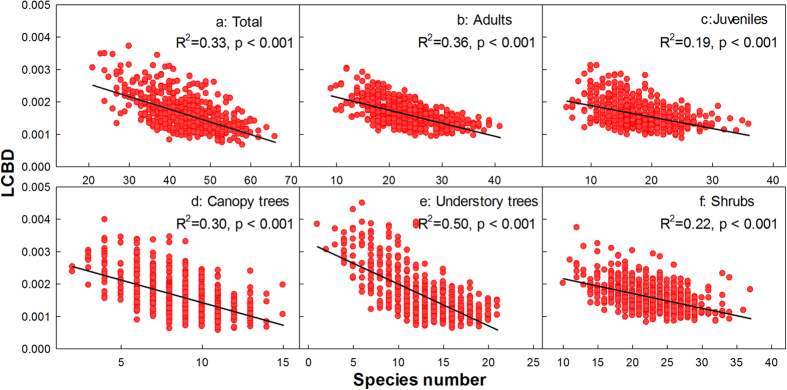
Relationships between local contribution to beta diversity (LCBD) and species richness. (**a**) Total species, (**b**) Adults, (**c**) Juveniles, (**d**) Canopy trees, (**e**) Understory trees, (**f**) Shrubs.

**Figure 5:**
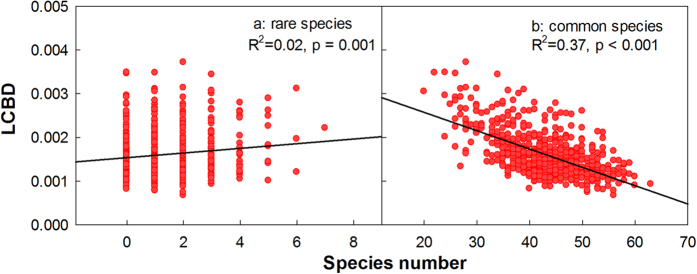
Relationships between local contribution to beta diversity (LCBD) and rare species richness (**a**) and common species richness (**b**).

LCBD was negatively related to species richness (*r* = –0.57, Fig. 4a), indicating that high LCBD values occurred when there were fewer species. Similar patterns were detected for different tree groups (Fig. 4b–f). Indeed, we found a positive relationship between LCBD and the number of rare species (Fig. 5a, *r* = 0.13, p < 0.001). Conversely, a significant negative relationship was found between LCBD and the number of common species (Fig. 5b, *r* = –0.61, p < 0.001).

## Discussion

Our analyses compared separately how the spatial and environmental factors influence the structure of various life stages and tree layers along with composition and beta diversity in the BDGS subtropical forest community. We found that a large portion of the variation of species composition (~60%) in the BDGS plot was jointly determined by environmental and spatial variables, similar to former studies in the Gutianshan^30^ (Zhejiang) and Lienhuachih^27^ (Taiwan) biodiversity plots. It is important to note that the undetermined variation was about 40% in all three studies, illustrating the effects of stochastic processes were about the same in different places. Differences existed in the proportion of variation explained by environment and space. In this study of the BDGS plot, spatial and environmental variables were both strongly related to species composition with space a bit stronger (environment 27% vs space 34%). Gilbert & Lechowicz found a stronger influence of environment relative to space on beta diversity in a low-diversity temperate forest understory^7^, while Condit *et al.* found that a purely spatial model predicted beta diversity well in tropical tree communities^18^. De Cáceres *et al.* found that spatial factors explained more variation in tropical forest plots than temperate forest plots^37^. Together, these results suggest that in high-diversity communities, neutral processes usually play a more important role than in low-diversity communities, e.g. those located in temperate regions. The BDGS plot is located in the northern subtropical region, and species richness is approximately midway between tropical and temperate regions. As a result, both environment and space variables were important factors related to species composition of this plot.

In order to be compare our study with former studies that only included topographic variables to examine environment (i.e., no soil variables), we reran the analysis including only topography, and the results showed that the environmental fraction had a much lower explanatory power than the spatial fraction (topography, 15.7%, vs spatial eigenfunctions, 44.7%). Our results were inconsistent with those of the Gutianshan plot, in which topographical and spatial variables equally accounted for the distribution of beta diversity (topography 30.7% vs spatial eigenfunctions 34.8%)^30^. However, our results were consistent with what was found in the Lienhuachih forest in Taiwan, in which topography alone explained 20.7% of the species composition while spatial variables explained 37.5%^27^. Chang *et al.* found that if only topography was included, dispersal-based processes prevailed, but including soil variables reversed this conclusion in favor of niche-based processes^27^. Our results illustrate that topography is indeed not sufficient to account for the environmental variation controlling species composition in the BDGS plot.

Community composition of adult trees was more weakly associated with measured habitat variables than juvenile composition. However, after removing sampling effects, there were no differences between the two groups. This opposes our expectation that adult trees may be more impacted by habitat filtering than juveniles^38^. One probable reason was the high densities of juveniles in their preferred habitat, while subsequent thinning caused by negative density dependence during the transition to reproductive individuals weakens the association of adults with habitat variables^39^. The process of removing sampling effects mirrored the effect of negative density dependence, so the strong relation of habitats with juvenile tree diversity and composition disappeared. These results may also suggest that the distribution of trees in different life stages are determined by common factors or that habitat filtering occurs in earlier stages (e.g., germination and seedling survival; all trees in this study were larger than 1 cm in DBH).

For the community composition of different tree layers, the contributions of environmental variables were about the same. However, shrubs had the highest explanation by spatial variables. After removing sampling effects, this difference disappeared, suggesting it was the result of larger numbers of individuals. After removing sampling effects, canopy trees had the highest explanation rates by environmental variables for community composition. Our results were consistent with Guèze *et al.*^33^ and Paoli *et al.*^40^; both found large tree patterns were explained more by environmental variables, suggesting an increasing importance of niche processes with increasing tree diameter class. However, Kristiansen *et al.* found understory palms of Peruvian Amazonia were more strongly controlled by environmental variables than canopy palms^20^. A probable reason for this difference may be again sampling effects. Before removing sampling effects, our results also showed a higher explanation percentage for shrubs.

In this study, we also calculated the local contribution to beta diversity (LCBD) and found that topographical and soil variables both affected beta diversity, which was consistent to the variation partitioning results and corroborate environment filtering’s effects on species composition in this forest. We also found there existed a positive relationship between LCBD and the number of rare species and a significant negative relationship was found between LCBD and the number of common species. This means plots with low diversity had high uniqueness in the BDGS plot, which may be the result of special ecological conditions that should be given more attention for conservation^41^. The optimal goal of conservation is to protect the maximum number of species and habitats with limited funds; hence, unique communities are important for optimizing conservation efforts and beta diversity may be as important as alpha diversity^42^.

The spatial component represents the role of dispersal limitation in cases where all relevant environmental variables are considered; otherwise, an unknown proportion of the spatial structure may be due to unmeasured environmental variables^43^. When partitioning species variation between spatial and environmental explanatory variables, some uncertainty remains as to the interpretation of the spatially-structured variation that is not explained by the topographic variables^44^; i.e., it is likely that we overestimated the pure spatial component and underestimated the environmental component. Such knowledge illustrates researchers should collect as many environmental variables as possible, including topography, soil, light, etc., to provide the most robust results. Results are trustworthy when environment prevails over space; however, in the opposite case, when space prevails over environment, results are not necessarily realistic. In our study, we found space and environment (based on topographic and soil variables) were both related to beta diversity. This result needs to be tested further in cases with more environmental variables. In addition, our approach assumed all species were independent, ignoring phylogenetic and functional differences between species. In future studies, the partitioning of phylogenetic and functional beta diversity may provide additional insight about the evolutionary and ecological processes that structure the BDGS communities^45^,^46^.

In conclusion, our study suggests that the diversity of the forest in BDGS plot is governed by both neutral and niche processes. Additionally, niche processes were more important for canopy trees than shrubs. However, patterns of adult and juvenile trees were in general congruent.

## Methods

### Location and description of the study plot

The 25-ha forest plot (500 m × 500 m; 29°45′54″-46′16″ N, 110°05′07″-05′25″ E) is located in the Badagongshan National Nature Reserve (232 km^2^ in area, 29°39′18″-29°49′48″ N, 109°41′50″-110°09′50″ E), Hunan Province, Central China. The reserve was set up in 1982 to preserve the mixed evergreen and deciduous broad-leaved forests and species extending for more than 300 ha in the region. Annual mean temperature in the region is 11.5 ^o^C and annual mean precipitation is 2105 mm. Forest covers 93.4% of the reserve area, and a total of 1996 species of seed plants belonging to 778 genera and 167 families have been inventoried thus far. *Fagus lucida* and *Cyclobalanopsis multinervis* are the dominant species. Altitude ranges from 1354.7 to 1455.9 m above sea level and slopes of the 20-m^2^ cells range from 4.3° to 72.2°.

### Tree data

The 25-ha plot was divided into 625 cells 20 m × 20 m in size. All trees with diameter at breast height (DBH) ≥ 1 cm were tagged, identified, measured and georeferenced between March of 2010 and November of 2011. In total, we recorded 186,556 individuals belonging to 53 families, 114 genera and 238 species in the plot. Species abundances ranged from 1 to 21,110 individuals. In order to maintain relative species abundance when removing effects of sample size, we only retained species with more than 10 individuals. Data matrices used in the analyses contained 186,310 individuals from 165 species. Counting the number of living trees of each species in every cell in the grid, we obtained for each forest plot an *n* × *p* (cell-by-species) data table *Y* = [y_*ij*_], y_*ij*_; the number of live individuals of species *j* in cell *i*.

We categorized trees into three growth forms (canopy trees, understory trees and shrubs) and two life stages (adults and juveniles) (Table 2). Generally, adults and juveniles were defined on the basis of tree size (DBH), although size (DBH) is not an exact measure of age^47^. We ranked the trees by DBH within each species, categorizing stems with DBH < DBH99^1/2^ (DBH99 represents the 99th percentile of DBH) as juveniles and stems with DBH ≥ DBH99^2/3^ as adults. Trees between DBH > DBH99^2/3^ and DBH < DBH99^1/2^ were excluded to accentuate the difference between classes^47^,^48^. The 99^th^ percentile was used to reduce the effects of outliers.

The number of individuals sampled for different groups varied enormously, and sample size is known to affect model accuracy^48^. In order to remove the effects of sample size, we randomly retained approximately the same number of individuals for each category (maintaining their relative species abundance) based on the category with the fewest individuals^29^. For example, for different life stages, there were 111, 869 individuals for juveniles but only 43, 075 individuals for adults. So we randomly drew approximately 43, 075 juvenile individuals from all juvenile individuals and repeated 10 times; the mean of these 10 runs was used for subsequent analyses.

### Environmental and spatial descriptors

We used topography and soil as a proxy of the microenvironmental conditions within plot cells^30^. Four topographic variables were used in this study: altitude, slope, aspect and convexity. Aspect refers to the direction to which a slope faces. Convexity is measured as the elevation of a cell of interest minus the mean elevation of the eight surrounding cells; for edge cells, convexity is the elevation of the centre point minus the mean of the four corners. We used altitude, convexity and slope to construct third-order polynomials for a total of nine monomials^37^. The circular variable *aspect* cannot be used as a predictor in linear models; we transformed it to annual direct incident radiation and heat load based on latitude, slope and aspect following McCune and Keon^49^ which can be used together in this type of model^30^. Soil samples were taken and analyzed for 13 parameters, including total C and N, C/N ratio, P, δ^13^C isotope of two soil layers (0–10 cm and 10–30 cm), pH value, bulk density, and C density of the first soil layer (0–10 cm). The specific sampling protocol can be found in Appendix S1. Based on the original soil data from 972 sampling points, we obtained soil variables for every 20 m × 20 m quadrat from Ordinary Kriging using Arc Map 10.1. The parameters of semi-variogram models for all soil properties were larger than our minimum sampling distance, so models could capture significant spatial variation in soil variables (Appendix S2). Third-degree polynomial equations were conducted for each soil variable, resulting in 50 variables for the expanded environmental data table. We used both topography and soil as environmental variables in most of our analyses, but retained topography separately in the variation partitioning of total trees in order to compare with former studies that did not include soil parameters.

Spatial structure was represented by distance-based Moran’s eigenvectors maps (dbMEM) derived from spectral decomposition of the spatial relationships among cells^30^. Eigenfunctions were computed across the 625 cells describing all spatial scales accommodated by the sampling design. We used the forward selection method to identify significant dbMEM eigenfunctions from all eigenvectors (altogether, 440 eigenvectors). The dbMEMs eigenvectors with positive eigenvalues model positive spatial correlation; for parsimony, only these positive eigenvectors were included in the data table of spatial descriptors S^50^.

### Statistical analysis

Hellinger distance was used to measure the dissimilarity in the species composition among plot cells in this study^35^. The measure of tree beta diversity used in this paper is the total variation in the **Y**’ table. Var(**Y**’) is SS(**Y**’)/(*n*–1), where SS(**Y**’) = SS_Total_ is the sum, over all species and all cells, of the squared deviations from the species means^13^,^37^. The maximum possible total variation value for Hellinger-transformed data is 1, which represents all sites having entirely different species composition.

We used variation partitioning by canonical redundancy analysis (RDA) to decompose the variation of tree beta diversity into fractions explained by the environmental (**E**) and spatial (**S**) variables^50^,^51^. We conducted variation partitioning using the **Y**’ table as the response matrix and environmental table **E** and spatial table **S** as explanatory matrices. Following this method, we divided tree beta diversity variation into four components: pure topographic variation fitted by topographic variables independent of spatial variables [a], spatially-structured topographic variation fitted by both topographic and spatial variables [b], pure spatial variation fitted by spatial variables only [c], and unexplained variation [d]. We used the adjusted R square metric 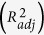 to represent the relative contribution of each component to overall variation. We interpreted component [a + b] as the variation explained by topographic variables (signifying niche processes) and component [c] as the variation explained by spatial variables (signifying neutral processes)^30^.

We also calculated the contributions of individual sampling units to the overall beta diversity, the local contribution to beta diversity (LCBD)^35^. We used multiple regressions to determine the relationships between LCBD and environmental variables (independent variables included third-degree polynomial equations). Former studies found a negative relationship between LCBD and species richness and indicated rare species combinations in species-poor areas^35^. Thus, we also identified rare species (those with fewer than 25 individuals) and common species in the plot, and ran separate regression analyses with LCBD for rare and common species.

The packages PCNM, “spacemakeR”, “packfor” and “vegan” in R were used to conduct the dbMEM, forward selection, RDA and variation partitioning. Total beta diversity and the derived indices were computed using the beta.div function^35^.

## Additional Information

**How to cite this article**: Qiao, X. *et al.* Beta diversity determinants in Badagongshan, a subtropical forest in central China. *Sci. Rep.* **5**, 17043; doi: 10.1038/srep17043 (2015).

## Supplementary Material

Supplementary Information

## Figures and Tables

**Table 1:** Relationships between LCBD (local contributions to beta diversity) and environmental variables.

Variables	R^2^	R_a_^2^	P value
Topography			
Altitude	0.151	0.147	<0.001
Slope	0.071	0.067	<0.001
Convexity	0.104	0.099	<0.001
Aspect	0.014	0.011	0.012
Soil features of the upper layer			
C	0.003	−0.002	0.634
δ^13^C	0.062	0.058	<0.001
N	0.005	−0.000	0.406
P	0.008	0.003	0.161
N/P	0.015	0.010	0.024
C density	0.001	−0.003	0.822
Bulk density	0.004	−0.001	0.489
pH	0.163	0.159	<0.001
Soil features of the lower layer			
C	0.004	−0.001	0.464
δ^13^C	0.024	0.019	0.002
N	0.028	0.023	0.001
P	0.042	0.039	<0.001
N/P	0.018	0.015	0.004

For each variable, the independent variables include third-degree polynomial equations.

**Table 2:** Number of species and individuals of different life stage and life form categories.

	Category	No. of species	No. of individuals
Life stages	Juveniles	164	111869
	Adults	165	43075
Life forms	Canopy species	33	33015
	Understory species	42	66525
	Shrubs	90	86770
