# Supplementary material for: Beta diversity determinants in Badagongshan, a subtropical forest in central China
Source: Sci Rep. 2015 Nov 23;5:17043. doi: 10.1038/srep17043 (PMC4655473; doi:10.1038/srep17043)
Supplement: Supplementary Information [file srep17043-s1.pdf]

Beta diversity determinants in Badagonshan, a subtropical forest in central China

Xiujuan Qiao, Qianxi Li, Qinghu Jiang, Junmeng Lu, Scott Franklin, Zhiyao Tang,Qinggang Wang, Jiaxin Zhang, Zhijun Lu, Dachuan Bao, Yili Guo, Haibo Liu, Yaozhan Xu, Mingxi Jiang

Supplementary information

Appendix S1: Field sampling and laboratory analysis of soil

Appendix S2: Univariate statistics and parameters for the spherical semivariogram model of soil properties

Appendix S1

Field sampling and laboratory analysis of soil

We sampled 972 points using both regular and random sampling techniques to cover the entire BDGS plot from June to September, 2013 (Robertson et al., 1997; Zhang et al. 2011). The main part (480m×480m in the bottom left corner) of the plot was divided into 30m×30m quadrats. The remaining area was divided into 32 20m×30m quadrats and 1 20m×20m quadrat. Soils were sampled at each crossing point. In order to capture variation in soil properties at finer scales, two of three distances (2 m, 5 m, and 15 m) were randomly chosen along a random compass direction from each crossing point. All sampling points were kept within the 25-ha plot (Figure.S1). The smallest distance between points was 1.22 m and the max was 707.12 m. Thus, we sampled soils in two layers (0-10cm and 10-30cm) using a 3.4cm diameter soil auger. Three subsamples within 0.2m of the sample location were obtained after the litter layer was removed and subsequently mixed for laboratory analysis.

All soil samples were air-dried, then passed through 2.0 and 0.15mm sieves. For each soil sample, soil pH was measured in 1M KCL solution (soil : solution ratio of 1:2.5). Soil organic carbon, total nitrogen and $\delta^{13}C$ were determined using dry combustion methods on an elementary analyzer - stable isotope ratio mass spectrometer (EA-IRMS, Thermo Scientific Flash 2000 HT and Delta V Advantage, German). Soil available phosphorus was extracted with 0.03mol/L NH_4F-0.1mol/L HCl solution, and then determined using colorimetry on a TECAN Infinite M200pro.

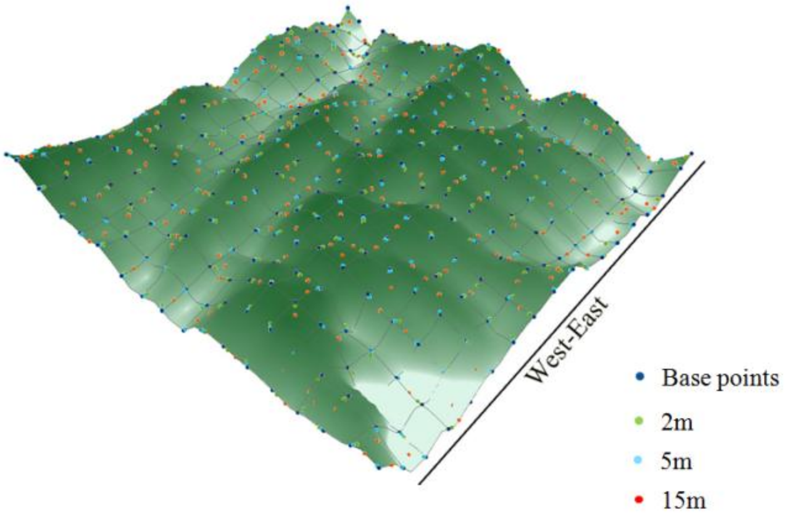

Figure.S1 Distribution of the 972 soil sampling points in BDGS plot.

References:

Robertson G.P. *et al*. Soil resources, microbial activity, and primary production across an agriculture ecosystems. Ecol. Appl 7: 158-170. (1997).

Zhang, L.W., Mi, X.C., Shao, H.B.,Ma, K.P. Strong plant-soil association in a heterogeneous subtropical broad-leaved forest. Plant and Soil. 347, 211-220. (2011).

Appendix S2

Table S1 Univariate statistics and parameters for the spherical semivariogram models of soil properties.

	unit	mean	SD	CV	Range(m)	C	C_0	C/ C_0+C
$SOC_{0\text{-}10cm}$	%	8.42	2.61	0.31	29.21	0.01	0.03	0.31
$SOC_{10\text{-}30cm}$	%	4.71	1.46	0.31	29.46	0.04	0.06	0.42
$STN_{0\text{-}10cm}$	%	0.63	0.17	0.27	15.84	0.02	0.05	0.31
$STN_{10\text{-}30cm}$	%	0.34	0.1	0.3	27.03	0.02	0.02	0.47
pH	-	4.56	0.37	0.08	48.73	0.09	0.04	0.69
$AP_{0\text{-}10cm}$	mg/kg	6.49	4.62	0.71	12.97	0.22	0.71	0.24
$AP_{10\text{-}30cm}$	mg/kg	2.58	2.51	0.97	160	0.09	0.75	0.1
$\delta^{13}C_{0\text{-}10cm}$	-	-27.1	0.61	0.02	31.6	12.82	23.28	0.36
$\delta^{13}C_{10\text{-}30cm}$	-	-25.85	0.71	0.03	52.4	2.31E+08	2.58E+08	0.47
Bulk density	g/cm^3	0.58	0.16	0.27	52.66	0.01	0.02	0.32

C represents Nugget; C_0 represents Sill.
